# Clinical features of neuronal intranuclear inclusion disease with seizures: a systematic literature review

**DOI:** 10.3389/fneur.2024.1387399

**Published:** 2024-04-19

**Authors:** Jinwei Zhang, Ling Ling, Lei Xiang, Wenxia Li, Pengnan Bao, Wei Yue

**Affiliations:** ^1^Clinical College of Neurology, Neurosurgery and Neurorehabilitation, Tianjin Medical University, Tianjin, China; ^2^Department of Neurology, Tianjin Huanhu Hospital, Tianjin, China

**Keywords:** neuronal intranuclear inclusion disease, seizure, diagnosis, prognosis, NOTCH2NLC gene

## Abstract

**Background:**

Infant, junior, and adult patients with neuronal intranuclear inclusion disease (NIID) present with various types of seizures. We aimed to conduct a systematic literature review on the clinical characteristics of NIID with seizures to provide novel insight for early diagnosis and treatment and to improve prognosis of these patients.

**Methods:**

We used keywords to screen articles related to NIID and seizures, and data concerning the clinical characteristics of patients, including demographic features, disease characteristics of the seizures, treatment responses, imaging examinations, and other auxiliary examination results were extracted.

**Results:**

The included studies comprised 21 patients with NIID with seizures. The most common clinical phenotypes were cognitive impairment (76.20%) and impaired consciousness (57.14%), and generalized onset motor seizures (46.15%) represented the most common type. Compared with infantile and juvenile cases, the use of antiepileptic drugs in adults led to significant seizure control and symptom improvement, in addition to providing a better prognosis. The number of GGC sequence repeats in the NOTCH2NLC gene in six NIID patients with seizures who underwent genetic testing ranged 72–134.

**Conclusion:**

The most common clinical phenotypes in patients with NIID with seizures were cognitive impairment and consciousness disorders. Patients with NIID presented with various types of seizures, with the most common being generalized onset motor seizures. Adult patients had a better prognosis and were relatively stable. The early diagnosis of NIID with seizures is of great significance for treatment and to improve prognosis.

## Introduction

Neuronal intranuclear inclusion disease (NIID) is a rare and slowly progressing neurodegenerative condition characterized by the presence of transparent eosinophilic inclusion bodies in the cells of the central, peripheral, and autonomic nervous systems and visceral organs (1–4). NIID can be divided into child, adolescent, and adult types based on the age and course of onset. The adult type can be further divided into sporadic and familial types based on family genetics (5, 6). The clinical manifestations of NIID are heterogeneous and involve the central, peripheral, and autonomic nervous systems (7–10).

NIID displays eosinophilic transparent inclusion bodies in adipocytes, fibroblasts, and sweat gland cells based on skin biopsy, and diffusion-weighted imaging (DWI) in magnetic resonance imaging (MRI) examinations shows characteristic high signals at the corticomedullary junction, which can assist in diagnosis (11). Moreover, GGC repeat amplification in the 5′ region of the notch homolog 2 N-terminal-like protein C gene (NOTCH2NLC) is seen in NIID, enabling disease recognition and early diagnosis (2, 12–15). However, in some patients with atypical and nonspecific clinical manifestations, especially in those with no imaging-based manifestations, it is difficult to diagnose NIID based solely on clinical presentations.

NIID can present with slow progressive symptoms, such as dementia, Parkinson’s syndrome, cerebellar ataxia, peripheral neuropathy, and autonomic dysfunction (7, 8, 16, 17), as well as acute episodic symptoms such as consciousness disorders, episodic encephalopathy, and stroke-like seizures (8, 9, 17). This disease can also cause various types of seizures in infant, junior and adult patients (1, 18, 19). The area of epileptic lesions seen on electroencephalography (EEG) is consistent with the DWI hyperintensities (20), and areas with swelling and layered necrosis in the brain are also the areas with the strongest epileptic discharge (6). Thus, the cerebral cortex near the DWI hyperintense areas may be more active than other regions, which is likely related to the occurrence of seizures in these patients (21). However, the pathogenesis of seizures in NIID and the effect of seizures on NIID progression remain unclear. We, therefore, aimed to conduct a systematic literature review to determine the clinical characteristics of patients with NIID with seizures, and to discuss the possible mechanisms of NIID seizures to provide novel insights for early diagnosis and treatment as well as improving patient prognosis.

## Methods

### Search strategy and selection criteria

**Figure 1:**
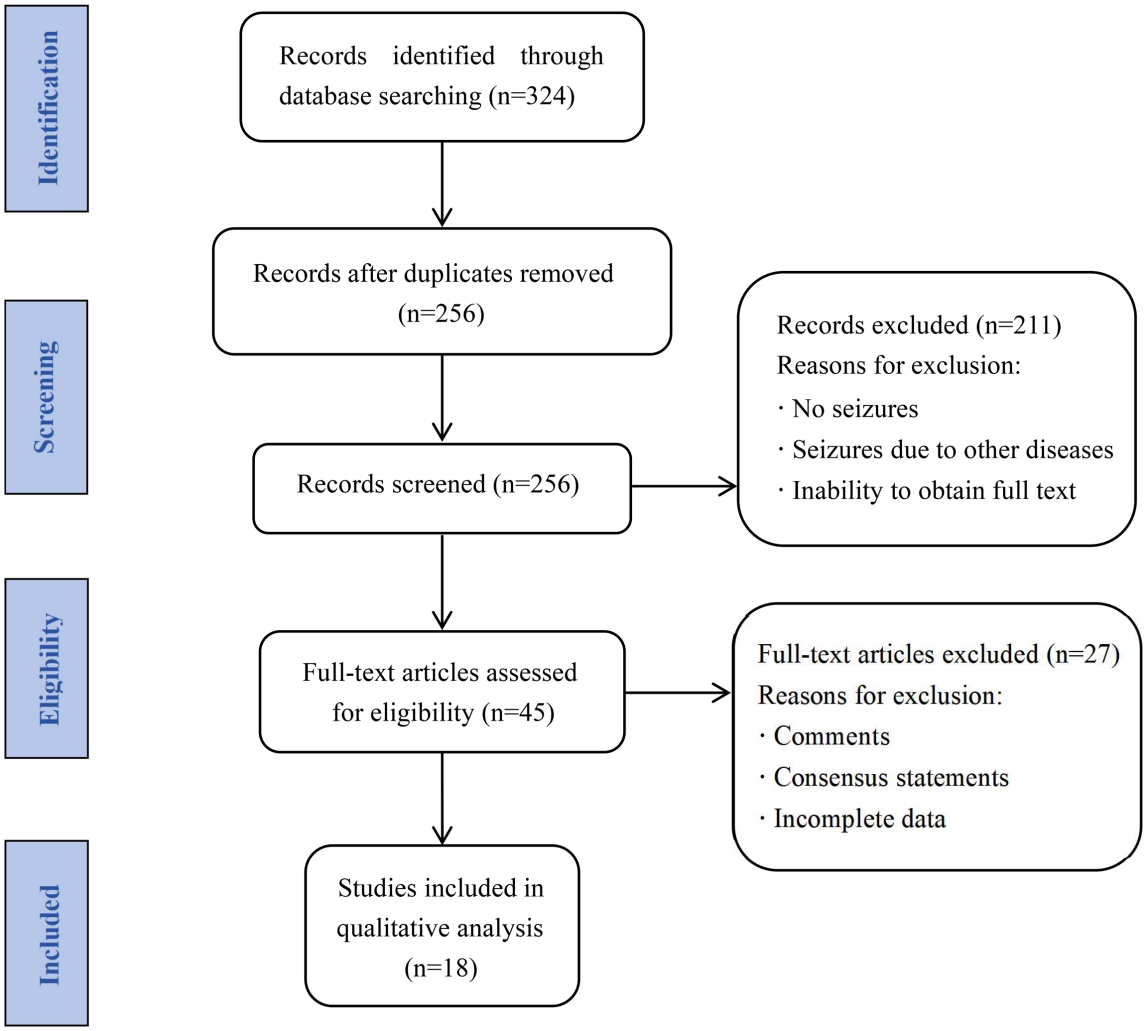
Flow chart of study selection.

We systematically searched the PubMed, Google Scholar, Wanfang, and China National Knowledge Infrastructure databases for studies related to NIID with seizures published between January 1984 and June 2023, using a combination of terms such as “neuronal intranuclear inclusion disease,” “neuronal intranuclear hyaline inclusion disease,” “seizure,” and “epilepsy.” The inclusion criteria were: (1) confirmed NIID based on clinical and imaging presentations, pathological testing, and genetic testing; and (2) seizures diagnosed based on clinical manifestations and EEG findings during the disease course. Studies with the following characteristics were excluded: (1) inclusion of seizures due to other diseases; (2) repeated cases; (3) comments and consensus statements; (4) incomplete demographic and clinical characteristic data; and (5) full text unobtainable. Case reports, case series, and retrospective and prospective observational studies were included in the analyses. After reviewing 324 articles and excluding repeated cases, studies comprising 556 patients with confirmed NIID were included. Eighteen articles were finally included, with a total of 21 cases, meeting all inclusion and exclusion criteria. The screening process is illustrated in Figure 1.

### Data extraction

Two reviewers conducted independent literature searches and extracted the following data for analysis: (1) demographic and disease-related characteristics, including age at onset, sex, disease course, family history, and clinical phenotype; (2) clinical features related to seizures, including clinical manifestations, seizure types, antiepileptic drugs, treatment effects, and EEG manifestations of seizures; and (3) results of auxiliary examinations and tests, including cranial MRI, cognitive function-related scores, nerve conduction velocity, blood creatine kinase (CK) levels, cerebrospinal fluid (CSF) biochemistry tests, tissue pathological biopsy, and genetic testing. The patients were divided into infantile, juvenile, and adult groups based on their age at onset and disease course. Seizure types were determined by two professional neurologists based on the 2017 International League Against Epilepsy (ILAE) classification (22).

### Data analysis

We used SPSS software (version 26.0; IBM Corp., Armonk, NY, United States) and GraphPad Prism (version 8.0; La Jolla, CA, United States) for statistical analysis and image rendering, respectively. Non-normally distributed econometric data are represented as medians and interquartile ranges (IQRs), whereas categorical data are represented as percentages (%). A descriptive quantitative analysis was conducted on the demographic data and disease characteristics of the patients. Differences in clinical phenotypes and seizure types were compared between patients with NIID with seizures at different ages. Classification variables were compared using Fisher’ s exact test. *p*-values <0.05 were considered statistically significant.

## Results

### Clinical characteristics of patients with NIID with seizures

After reviewing 324 studies and excluding repeated cases, 556 patients with confirmed NIID were identified, of which 39 (7.01%) experienced seizures. After screening, 18 observational studies (14 case reports and four cohort studies) from five countries met the inclusion criteria, with 12 published in English, two in Japanese, and four in Chinese. A total of 21 patients with NIID with seizures were included (11 from China, five from Japan, two from the United States, two from Canada, and one from Finland). The median age of onset in these cases was 59 (13–62) years, with no significant difference in patient sex (10 women and 11 men). Eight patients had a family history (8/17) of NIID.

Among the 21 included patients, two cases were infants, four were juveniles, and 15 were adults. There were five cases (5/20) with seizure as the initial symptom, and seven cases (7/20) with seizure as the main symptom. The other clinical manifestations of patients with NIID with seizures were heterogeneous, including various symptoms related to central, peripheral, and autonomic nervous system function. A detailed summary of the clinical features is provided in Table 1.

**Table 1:** Demographic and clinical characteristics of NIID cases with seizures.

Individual	1 (19)	2 (63)	3 (26)	4 (18)	5 (8)	6 (50)	7 (7)	8 (25)	9 (40)	10^NA^ (53)	11 (1)	12 (27)	13 (35)	14 (35)	15 (35)	16 (5)	17 (20)	18 (28)	19 (24)	20 (24)	21 (23)
Onset age	2.5	3.5	11	8	13	8	59	62	66	67	59	63	48	52	70	70	76	63	18	60	55
Sex (Female/male)	M	F	F	M	F	M	F	F	M	F	M	M	F	M	M	F	M	F	M	M	F
Disease duration (years)	2	3.25	10	14	7	12	14	5 M	6 M	20	4	4 M	3	3	1 M	2	3 M	0.5 M	12	2	1 M
Family history	+	−	+	NA	NA	+	+	NA	NA	−	−	−	−	+	−	−	+	+	−	−	+
Clinical manifestation																					
Central nervous system																					
Fever	−	−	−	−	−	−	−	+	−	−	+	+	−	−	−	−	+	−	+	−	−
Headache	−	−	−	−	−	+^1*^	−	−	−	−	+	−	−	−	+	−	−	−	+^1*^	+^1*^	+
Dizziness	−	−	−	−	−	−	+	+	−	−	−	−	+	+	−	−	−	−	−	−	−
Cognitive dysfunction	−	+	−	+	+	+	+	+	+^1*^	−	+	+^1^	+^1*^	+	+	+	+	−	+	+	−
Mental and behavioral abnormalities	−	−	+	+	+	−	−	+	+	−	−	+	−	−	+	−	−	+^1*^	+	−	+^1*^
Aphasia	+	−	−	−	−	+	−	+	+	−	+	−	−	−	−	−	−	−	−	−	+
Paroxysmal encephalopathy																					
Consciousness disorder	+	+	−	−	−	+	+	+	+	−	+	+	−	−	−	+	+	+	−	−	+
Seizure	+	+	+^1*^	+	+	+	+^*^	+	+	+	+	+^*^	+	+^1*^	+^1*^	+^1*^	+^1*^	+	+	+	+
Encephalitic episodes	−	−	−	−	−	−	−	+^1*^	−	−	−	+	−	−	−	−	+	−	−	−	−
Stroke-like episodes	−	−	−	−	−	+	−	−	−	−	+^1*^	−	−	−	−	−	−	−	−	−	−
Sensory disturbance	−	+	−	−	−	+	+	−	−	+	+	−	+	−	−	−	−	−	−	−	−
Muscle weakness	−	+	+	−	−	+	−	−	−	+	+	−	−	−	−	−	+	−	−	+	−
Dystonia	+	+^1*^	+	+^1*^	+^*^	−	−	+	−	−	−	−	−	−	−	−	−	−	−	−	−
Ataxia	+^1*^	+	+	−	+	−	−	−	−	−	−	+	+	−	−	−	−	−	−	−	−
Tremor	+	−	+	+	+^1^	−	−	−	−	+	−	−	−	−	−	+	−	−	+	−	−
Peripheral nervous system																					
Vision disorder	+	−	+	−	−	+	−	+	−	−	+	−	+	+	−	−	−	−	−	−	−
Polyneuropathy	−	+	−	−	−	−	−	−	−	−	−	−	−	−	−	−	−	−	−	−	−
Autonomic nervous system																					
Vomiting	−	−	−	−	−	+	+	−	−	−	−	+	+	−	−	−	−	−	−	−	−
Bladder dysfunction	+	−	−	−	−	−	+^1^	+	−	+	−	−	−	−	+	−	−	−	−	−	−
Miosis	−	−	−	−	−	−	−	−	−	+	−	+	−	−	−	−	+	+	−	−	−
Syncope	−	−	−	−	−	−	−	−	−	−	−	−	−	−	−	−	−	−	−	−	−

NIID, neuronal intranuclear inclusion disease. – negative; + positive; NA, not available. *Indicating the main symptom of patients. ^1^Indicating the initial symptom of patients.

**Figure 2:**
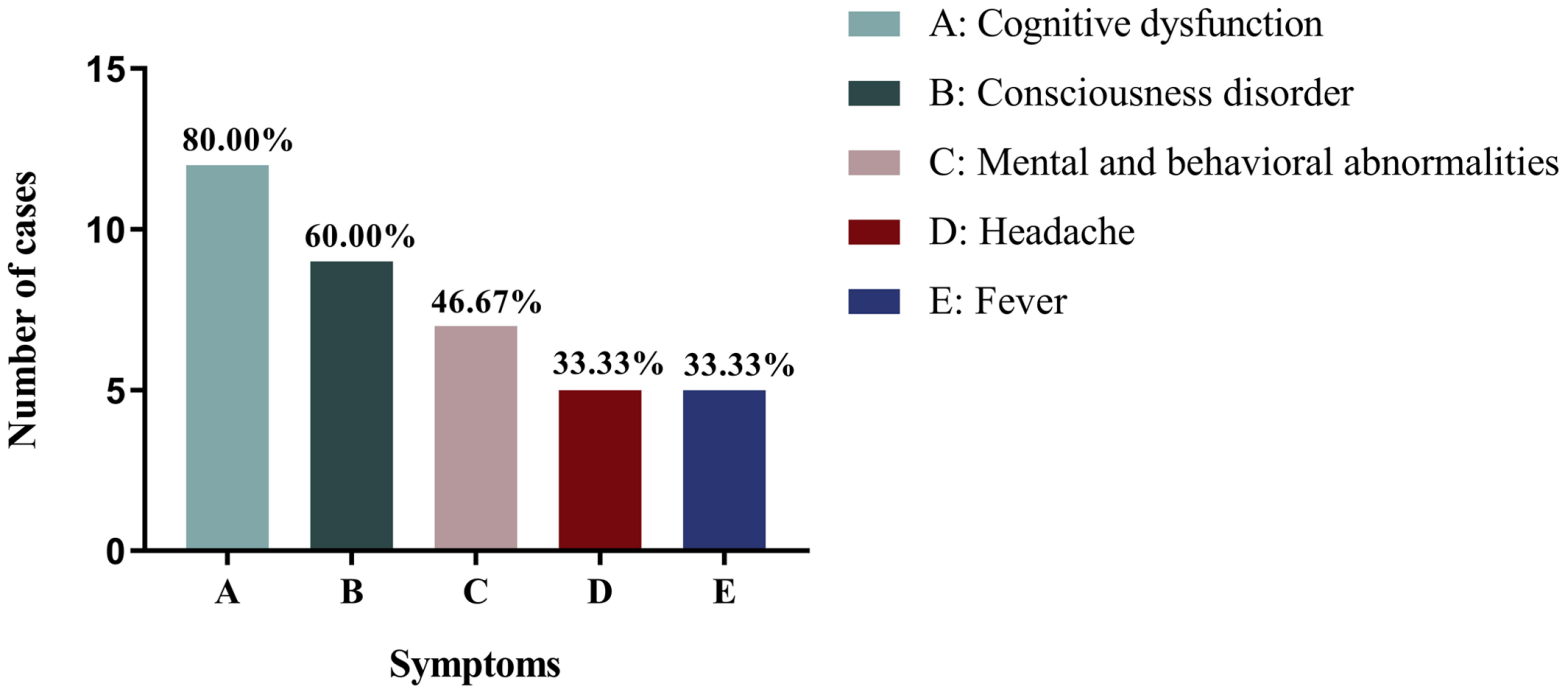
Different clinical phenotypes of adult NIID cases with seizures.

Among the other clinical phenotypes of patients with NIID with seizures, cognitive impairment was the most common, occurring in 16 cases (76.20%), followed by consciousness disorders (12 cases, 57.14%) and psychobehavioral abnormalities (10 cases, 47.62%). Infantile patients with NIID with seizures also experienced consciousness disorders (2/2), ataxia (2/2), and muscle tone disorders (2/2). Juvenile patients with NIID often showed cognitive impairment (3/4), psychobehavioral abnormalities (3/4), muscle tone disorders (3/4), and tremors (3/4). The other most common clinical manifestations in adult patients with NIID with seizures were cognitive dysfunction (80%), consciousness disorders (60%), and psychobehavioral abnormalities (46.67%; Table 2; Figure 2).

**Table 2:** Comparison of clinical characteristics at different ages of NIID cases with seizures.

Clinical characteristics	Total patients (*n* = 21)	Infantile form (*n* = 2)	Junvenile form (*n* = 4)	Adult form (*n* = 15)
Onset age, years, median (IQR)	59 (13–63)	3 (2.75–3.5)	9.5 (8–11.5)	62 (57–66.5)
Sex, Female, n (%)	10 (47.62)	1 (50.00)	2 (50.00)	7 (46.67)
Fever, n (%)	5 (23.8)	0 (0.00)	0 (0.00)	5 (33.33)
Headache, n (%)	6 (28.57)	0 (0.00)	1 (25.00)	5 (33.33)
Dizziness, n (%)	4 (19.05)	0 (0.00)	0 (0.00)	4 (26.67)
Cognitive dysfunction, n (%)	16 (76.20)	1 (50.00)	3 (75.00)	12 (80.00)
Mental and behavioral abnormalities, n (%)	10 (47.62)	0 (0.00)	3 (75.00)	7 (46.67)
Aphasia, n (%)	6 (28.57)	1 (50.00)	1 (25.00)	4 (26.67)
Consciousness disorder, n (%)	12 (57.14)	2 (100.00)	1 (25.00)	9 (60.00)
Encephalitic episodes, n (%)	3 (14.29)	0 (0.00)	0 (0.00)	3 (20.00)
Stroke-like episodes, n (%)	2 (9.52)	0 (0.00)	1 (25.00)	1 (6.67)
Sensory disturbance, n (%)	6 (28.57)	1 (50.00)	1 (25.00)	4 (26.67)
Muscle weakness, n (%)	7 (33.33)	1 (50.00)	2 (50.00)	4 (26.67)
Dystonia, n (%)	6 (28.57)	2 (100.00)	3 (75.00)	1 (6.67)
Ataxia, n (%)	6 (28.57)	2 (100.00)	2 (50.00)	2 (13.33)
Tremor, n (%)	7 (33.33)	1 (50.00)	3 (75.00)	3 (20.00)
Vision disorder, n (%)	7 (33.33)	1 (50.00)	2 (50.00)	4 (26.67)
Polyneuropathy, n (%)	1 (4.76)	1 (50.00)	0 (0.00)	0 (0.00)
Vomiting, n (%)	4 (19.05)	0 (0.00)	1 (25.00)	3 (20.00)
Bladder dysfunction, n (%)	5 (23.8)	1 (50.00)	0 (0.00)	4 (26.67)
Miosis, n (%)	4 (19.05)	0 (0.00)	0 (0.00)	4 (26.67)
Syncope, n (%)	0 (0.00)	0 (0.00)	0 (0.00)	0 (0.00)

NIID, neuronal intranuclear inclusion disease; IQR, interquartile range.

### Disease-related characteristics of seizures in patients with NIID

**Figure 3:**
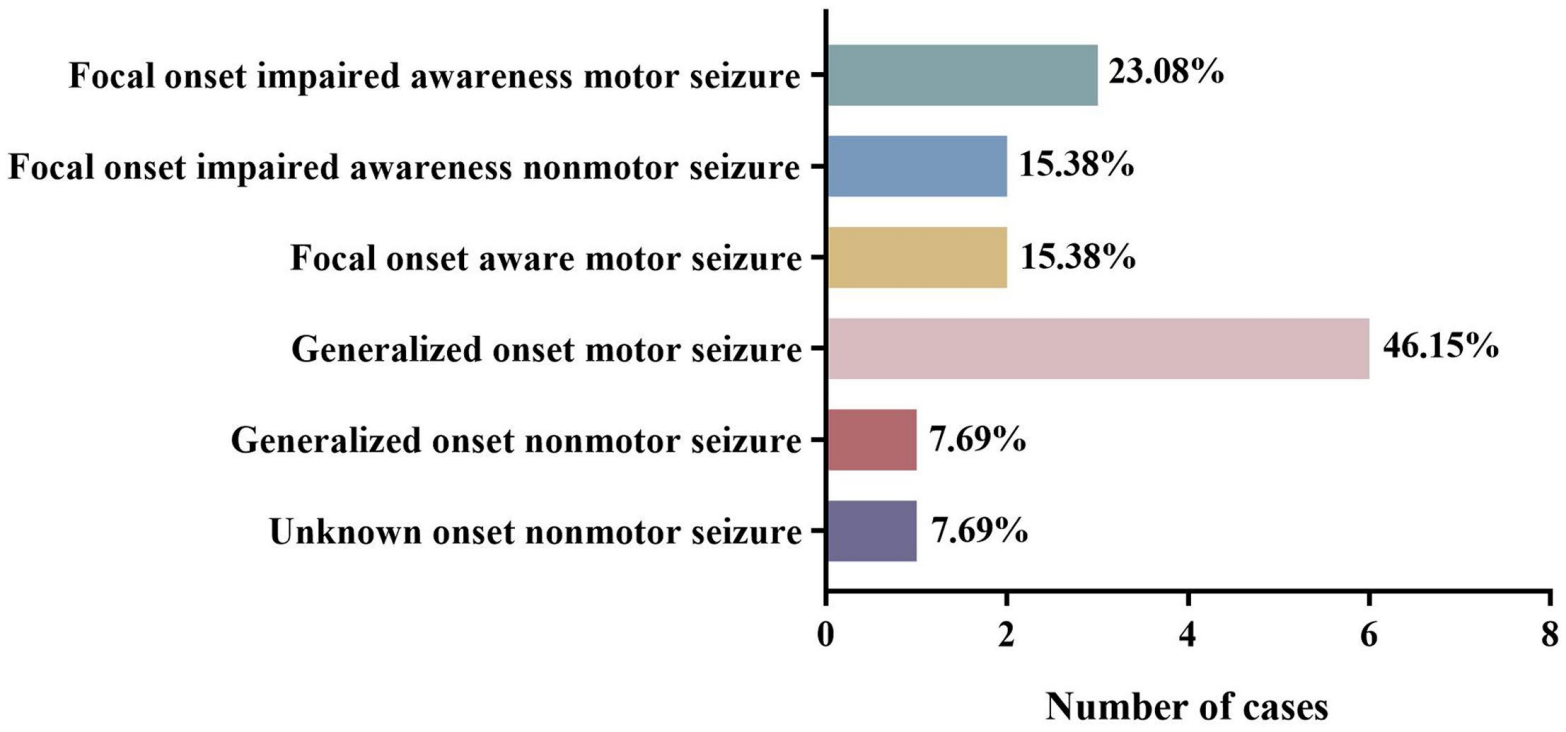
Different seizure types of NIID cases.

The included cases of NIID exhibited a variety of seizure types. Among the 13 cases with complete data, the most common seizure type was generalized motor seizure (6/13), accounting for 46.15%, including tonic–clonic seizures (4/6) and myoclonic seizures (2/6). Focal onset impaired awareness motor seizures accounted for 23.08% (3/13) of cases, including automatism (2/3) and other types of motor seizures (1/3). Focal onset impaired awareness nonmotor seizures (2/13) and focal onset aware motor seizures (2/13) each accounted for 15.38% of all cases. Other cases also presented with unknown onset nonmotor seizures (1/13) and generalized onset nonmotor seizures (1/13; Tables 3, 4; Figure 3). Among these 13 cases, three developed convulsive status epilepticus (CSE; 1/13) and one developed non-convulsive status epilepticus (NCSE; 1/13). One or more antiepileptic drugs were used, including levetiracetam (6/11), phenytoin sodium (4/11), carbamazepine (3/11), valproic acid (2/11), phenobarbital (1/11), diazepam (1/11), oxazepine (1/11), clonazepam (1/11), and topiramate (1/11). The EEG manifestations included epilepsy-like discharges (12/17), manifesting as sharp spikes in various parts (Table 3). Among the 14 cases with complete data, the prognosis of infantile and juvenile cases was poor, with four cases gradually progressing to death (4/6). No significant statistical difference was found in the mortality rate between the two groups (*p >* 0.05). Compared with infantile and juvenile cases, the use of antiepileptic drugs in adult patients significantly controlled and improved symptoms, and patient condition was stable (*p* = 0.010; Table 5).

**Table 3:** Clinical characteristics related to seizures of the cases.

Case	Clinical manifestation	Seizure types	Antiepileptic drugs	Curative effect	EEG
1 (19)	Myoclonic and absence seizures, consciousness disorder, CSE	Generalized onset motor seizure (myoclonic seizure), Generalized onset nonmotor seizure (absence seizure)	NA	Poor treatment effect, gradually worsening condition, and ultimately death	Initial EEG at age 2.5 years demonstrated generalized spike-and-wave activity and later disclosed generalized slow waves
2 (63)	NA	NA	NA	Poor treatment effect, gradually worsening condition, and ultimately death	Diffuse and later bursts of slow activity
3 (26)	Upward-turned gaze (oculogyric spasms) and tongue protrusion, periodic myoclonic jerks	Focal onset aware motor seizure, Generalized onset motor seizure (myoclonic seizure)	Phenytoin; Carbamazepine	Poor treatment effect, gradually worsening condition, and ultimately death	A mild abnormality, with paroxysms of slow waves of subcortical origin and general slowing of the background activity. Defined by the dominant rhythm, changed from mild to moderate abnormality, but the number of paroxysms was variable, with no definite trend
4 (18)	NA	NA	NA	Gradually worsening condition, and ultimately death	Generalized spike-and-wave discharges
5 (8)	Involuntary upward gaze, subtle mood changes	Focal onset impaired awareness motor seizure (automatism seizure)	NA	Gradually worsening condition	EEG showed abnormal, generalized epileptiform discharges with intermixed 1–2-s high-voltage slow waves with no clinical correlation and no electrographic seizures. Prolonged EEG monitoring showed bursts of bifrontal-predominant, spike-and-wave epileptiform activity that did not increase in frequency during sleep
6 (50)	Generalized tonic–clonic seizures, CSE, myoclonic epilepsy of the left extremities	Generalized onset motor seizure (tonic clonic seizure, myoclonic seizure)	Levetiracetam; Topiramate; Clonazepam	Decreased seizure attacks but frequent recurrence of myoclonic epilepsy of the left extremities	(1) Obviously asymmetrical hemispheres, with significantly reduced right amplitude and missing αrhythm and sleep wave; (2) irregular and unstable α rhythm at the left apex and occipital area, poor amplitude modulation, and partial α rhythm frequency slowed to 8 Hz; (3) sleep cycle staging was not obvious; (4) a paroxysmal tip-slow integrated wave group present in the central area in the midline; and (5) altered frequency of the spine-slow integrated wave in the right forehead, frontal, forehead, and midline areas
7 (7)	NCSE, consciousness disorder, no motor signs	Unknown onset nonmotor seizure	Phenytoin	Consciousness cleared, symptoms improved, but died 2 years later due to inhalation pneumonia	Generalized bilateral high amplitude periodic delta waves and sharp waves at 0.5–1 s intervals
8 (25)	Unconscious chewing, pouting, and other movement, consciousness disorder	Focal onset impaired awareness motor seizure (automatism seizure)	Levetiracetam; Valproic acid sodium	Symptom control but memory decline	Moderate abnormality, with generalized bilateral sharp waves and slow waves mainly in the posterior head, no α rhythm present
9 (40)	Limb convulsions, consciousness disorder	Generalized onset motor seizure	None	NA	None
10 (54)	NA	NA	NA	NA	NA
11 (1)	Episodic hemiplegia and consciousness disorder, no convulsion	Focal onset impaired awareness motor seizure	Phenytoin; Carbamazepine; Levetiracetam	Consciousness level improved, no major episodes for 1 year and 4 months after taking levetiracetam	Suppressed activity but no epileptic discharge in the left hemisphere
12 (30)	A transient episodic convulsion and alleviated after 20 s, no consciousness disorder	Focal onset aware motor seizure	Rehydration, and nutritional support therapy	Symptoms improved and condition stabilized	None
13 (35)	NA	NA	NA	NA	NA
14 (35)	NA	NA	NA	NA	NA
15 (35)	NA	NA	NA	NA	NA
16 (5)	Sudden loss of consciousness, no convulsion	Focal onset impaired awareness nonmotor seizure	Levetiracetam	Symptoms improved	The first interictal EEG showed a right mid-temporal spike; the second EEG showed sharp waves in the left central electrodes
17 (6)	Limb convulsions, consciousness disorder, CSE	Generalized onset motor seizure (tonic clonic seizure)	Diazepam; Carbamazepine; Phenytoin	Symptoms improved and condition stabilized	Generalized spikes, sharp waves, and slow waves in both frontal and left parietal lobes
18 (28)	Behavioral abnormalities, mild consciousness impairment, no convulsion	Focal onset impaired awareness nonmotor seizure	Levetiracetam	Symptoms improved, no recurrence within 8 months	Left posterior area spike wave/bilateral frontal area intermittent triangular wave
19 (24)	NA	NA	NA	NA	Widely intermittent complex slow wave, frequent sharp slow complex wave, and three-phase sharp wave in the right posterior head
20 (24)	NA	NA	NA	NA	Boundary EEG
21 (23)	Limb convulsions, episodic consciousness disorder	Generalized onset motor seizure (tonic clonic seizure)	Levetiracetam; Oxcarbazepine; Magnesium valproate; Phenobarbital	Epilepsy symptom control but persisting speech and behavior abnormalities	Epileptiform discharge on the left and right forehead, diffuse slow wave, with alternating dominance between the left and right frontal regions, and no sleep physiological waves observed

CSE, convulsive status epilepticus; EEG, electroencephalogram; NCSE, non-convulsive status epilepticus.

**Table 4:** Comparison of seizure types at different ages of NIID cases with seizure.

Seizure types (%)	Total patient (*n* = 13)	Infantile and juvenile form (*n* = 4)	Adult form (*n* = 9)
Focal onset impaired awareness motor seizure	3 (23.08)	1 (25.00)	2 (22.22)
Focal onset impaired awareness nonmotor seizure	2 (15.38)	0 (0.00)	2 (22.22)
Focal onset aware motor seizure	2 (15.38)	1 (25.00)	1 (11.11)
Generalized onset motor seizure	6 (46.15)	3 (75.00)	3 (33.33)
Generalized onset nonmotor seizure	1 (7.69)	1 (25.00)	0 (0.00)
Unknown onset nonmotor seizure	1 (7.69)	0 (0.00)	1 (11.11)

**Table 5:** Comparison of prognosis at different ages of NIID cases with seizure.

Outcomes (%)	Infantile and juvenile form (*n* = 6)	Adult form (*n* = 8)	*p*
Death	4 (66.67)	1 (12.50)	0.091
Symptoms improved	0 (0.00)	7 (87.50)	0.010*

*p < 0.05.

### Imaging and other auxiliary examinations in patients with NIID with seizures

The MRI examinations of all 15 adult patients with NIID with seizures showed high signal hyperintensities at the corticomedullary junction on DWI and white matter lesions on T2-weighted imaging (T2WI)/fluid attenuated inversion recovery (FLAIR) images. Some patients had cerebral atrophy (13/21), ventricular enlargement (7/21), or DWI signal hyperintensity in the corpus callosum (2/18).

In other auxiliary examinations, some patients showed abnormal neuropsychological scores, including Frontal Assessment Battery (FAB), Mini-Mental State Examination (MMSE), Montreal Cognitive Assessment (MoCA), Hamilton Anxiety Rating Scale (HAMA), and Hamilton Depression Rating Scale (HAMD) scores. Some patients also experienced decreased motor and sensory nerve conduction velocities (9/14). In CSF examinations, some patients showed a slight increase in CSF protein levels (6/15), but no significant increases in cell counts were observed. On pathological examinations, 20 of the 21 patients showed characteristic eosinophilic inclusion bodies in the nucleus on skin biopsy. Genetic testing revealed abnormal repetitive amplification of GGC sequences in the *NOTCH2NLC* gene of NIID patients. Six patients with NIID with seizures who underwent genetic testing showed 72–134 GGC sequence repeats (Table 6).

**Table 6:** Imaging examinations and other auxiliary examination results.

Individual	1	2	3	4	5	6	7	8	9	10	11	12	13	14	15	16	17	18	19	20	21
Head-MRI																					
T2WI/Flair Leukoencephalopathy	NA	NA	NA	−	−	+	+	+	+	+	+	+	+	+	+	+	+	+	+	+	+
DWI U-fiber high signals	NA	NA	NA	−	−	+	+	+	+	+	+	+	+	+	+	+	+	+	+	+	+
Brain atrophy	+	+	+	−	+	−	+	−	+	−	−	+	+	+	+	−	+	−	+	−	+
Ventricle dilation	+	+	+	−	−	−	−	−	+	−	−	−	−	+	−	−	+	−	+	−	−
DWI high signals in splenium of corpus callosum	NA	NA	NA	−	−	+	−	−	−	−	−	−	−	−	−	−	−	−	+	−	−
Executive function tests																					
FAB	NA	NA	NA	NA	NA	NA	NA	NA	NA	NA	11	NA	NA	NA	NA	18	NA	18	NA	NA	NA
MMSE	NA	NA	NA	NA	NA	NA	NA	23	NA	NA	25	NA	NA	NA	NA	29	NA	25	NA	NA	NA
MoCA	NA	NA	NA	15	NA	NA	NA	21	NA	NA	NA	NA	NA	NA	NA	NA	NA	NA	NA	NA	NA
HAMA	NA	NA	NA	NA	NA	NA	NA	13	NA	NA	NA	NA	NA	NA	NA	NA	NA	NA	NA	NA	NA
HAMD	NA	NA	NA	NA	NA	NA	NA	16	NA	NA	NA	NA	NA	NA	NA	NA	NA	NA	NA	NA	NA
Nerve conduction																					
MCV slowing	−	NA	−	−	−	+	+	+	NA	NA	+	NA	+	+	NA	−	NA	−	+	+	NA
SCV slowing	−	NA	−	−	−	+	+	+	NA	NA	+	NA	+	+	NA	+	NA	−	+	+	NA
Laboratory data																					
Serum CK (U/L)	NA	NA	161	−	NA	NA	NA	NA	NA	NA	209	NA	NA	NA	NA	NA	NA	NA	NA	NA	NA
CSF																					
Cell (*10^6^/L)	NA	NA	−	NA	−	−	NA	−	NA	NA	−	−	−	NA	−	2	NA	−	−	−	NA
Protein (mg/L)	−	−	−	NA	−	−	NA	866	NA	NA	−	720	520	NA	−	494	840	−	470	−	NA
Glucose (mmol/L)	NA	NA	−	NA	−	−	NA	−	5.4	NA	−	−	NA	NA	NA	NA	8.0	−	−	−	NA
Chloride (mmol/L)	NA	NA	−	NA	−	−	NA	−	107.8	NA	−	−	NA	NA	NA	NA	NA	−	−	−	NA
Intranuclear Inclusions	+	+	+	+	+	+	+	+	+	+	+	+	−	+	+	+	+	+	+	+	+
GGC repeat size	NA	NA	NA	NA	NA	NA	NA	NA	NA	NA	NA	72	134	97	81	NA	NA	NA	113	128	NA

MRI, magnetic resonance imaging; T2WI, T2-weighted images; FLAIR, fluid attenuated inversion recovery; DWI, diffusion-weighted imaging; MCV, motor nerve conduction velocity; SCV, sensory nerve conduction velocity; FAB, Frontal Assessment Battery; MMSE, Mini-mental State Examination, MoCA, Montreal Cognitive Assessment; HAMA, Hamilton Anxiety Scale; HAMD, Hamilton Depression Scale; CK, creatine kinase; CSF cerebrospinal fluid. – negative; + positive; NA, not available.

## Discussion

The present systematic review included reports on 21 patients with NIID with seizures, revealing that the most common clinical phenotypes of patients with NIID with seizures were cognitive dysfunction and consciousness disorders, with significantly decreased cognitive function after seizures in some cases. Additionally, patients with NIID exhibited various types of seizures, most commonly generalized onset motor seizure; compared with infantile and juvenile patients with NIID, adults had a better prognosis, more controlled seizure symptoms, and relatively stable condition.

The clinical manifestations of NIID include slow progressive symptoms related to dementia, Parkinson’s disease, cerebellar ataxia, peripheral neuropathy, and autonomic dysfunction (4, 7, 8, 16, 17), as well as acute episodic symptoms such as consciousness disorders, episodic encephalopathy, and stroke-like seizures (8, 9, 17). NIID is associated with various types of seizures in children, adolescents, and adult patients (23–28). In the present review, the median age of onset for patients with NIID with seizures was 59 years, with no significant sex differences. Most of the 21 included patients were adults. Five and seven patients presented with seizure as the initial and main symptom, respectively. Patients with NIID with seizures also exhibit heterogeneity in other clinical manifestations, including symptoms of the central, peripheral, and autonomic nervous systems. The main manifestations of adult patients with NIID with seizures were impairments to cognitive function and consciousness. In previous studies, the common symptoms of limb weakness in adult patients with NIID only manifested in 26.67% of patients (9). Therefore, if patients experience significant cognitive impairments or consciousness disorders along with seizures, a diagnosis of NIID should be considered.

Patients with NIID with seizures may develop secondary brain injury (29) leading to neurological dysfunction (6, 27, 30). After clinical symptoms gradually recover, brain tissue edema and layered necrosis can remain in the areas with the strongest epileptic discharge, delaying the recovery of brain function (6, 31–33). In the present review, Case 15 experienced significant deteriorations in cognitive function after experiencing behavioral changes and seizures within 1 month of onset (34). White matter encephalopathy may be the main cause of cognitive impairment in adult NIID (8, 9, 35, 36). Thus, patients with adult-onset NIID may first experience white matter damage, followed by atrophy of the hippocampus and neocortex, leading to executive dysfunction, memory impairment, and continuous deterioration in cognitive abilities (9, 37). Seizures may exacerbate this pathological process (9, 12, 37, 38). In another study, one patient experienced seizure characterized by paroxysmal memory loss that returned to normal (34). Although the clinical manifestations were atypical, the diagnosis of NIID was confirmed using neuroimaging, skin biopsy, and genetic testing (34). In clinical practice, NIID should be excluded if patients experience unexplained rapid cognitive decline after seizures. Further research is required to investigate the impact of seizures on cognitive function in patients with NIID.

In this systematic review, NIID cases presented with focal, generalized, and multiple types of seizures of unknown origin, the most common being generalized tonic–clonic seizures. Moreover, when compared with infantile and juvenile patients with NIID with seizures, adults had a better prognosis, more controlled seizure symptoms, and relatively stable conditions. Case 7 showed slow cognitive deterioration accompanied by disordered consciousness. Although the patient showed no obvious motor seizures, EEG showed extensive periodic bilateral high-amplitude triangular waves and sharp waves with an interval of 0.5–1 s, which was confirmed to be NCSE. The symptoms of consciousness recovery improve with the use of antiepileptic drugs (7). Because of the possibility of progressive cognitive impairment, which makes it difficult to detect a loss of consciousness caused by NCSE in adult patients with NIID patients, the diagnosis of NCSE may be missed (7). In clinical practice, patients with NIID experience prolonged cognitive impairment and encephalitis-like episodes which may be related to seizures, including NCSE. Long-term continuous EEG monitoring can be used to promptly detect seizures promptly and provide appropriate symptomatic treatment to improve patient prognosis.

Regarding imaging manifestations, 15 adult patients showed signal hyperintensities in the dermomyelinal junction on DWI. The areas of epileptic lesions seen on EEG are consistent with the high-signal areas on DWI (20). EEG in Case 17 showed a low-amplitude fast wave starting from the left parietal lobe (C3) and gradually spreading to the bilateral cerebral hemispheres, with widespread spikes and sharp waves (6). T2WI showed diffuse white matter lesions in both frontal lobes, whereas DWI shows a high signal hyperintensities at the corticomedullary junction between the frontal and left parietal lobes (6). The epileptic lesion was consistent with the high-signal area, with brain swelling and layered necrosis appearing in the left parietal lobe, which was also the area with the strongest epileptic discharge (6). Thus, the cerebral cortex near the DWI signal hyperintensity may be more active than other regions, and this is associated with seizures, unconscious, and NCSE in patients with NIID (21).

The occurrence of seizures in patients with NIID may be closely related to changes in perfusion to the cerebral cortex and medulla. Arterial spin labeling (ASL) imaging in Case 9 showed decreased diffused white matter perfusion in both the cerebral cortex and subcortical regions (39). Case 11 showed differences in cerebral perfusion during focal epileptic seizure, with high and low perfusion during and after onset, respectively (1). During the first seizure, ASL and magnetic resonance angiography (MRA) showed ipsilateral hypoperfusion. During the second day, single-photon emission computed tomography (SPECT) showed significant hyperperfusion in the corresponding areas (1). In the second seizure, ASL and MRA showed hypoperfusion in the right posterior area. On the second day, the images showed significant hyperperfusion in the corresponding area (1). The changes in perfusion in this patient were similar to those seen in migraine with aura, although the patient did not experience headache symptoms throughout the disease duration (1). In migraine with aura, changes in cerebral blood flow are biphasic, with low perfusion followed by high perfusion. Among the 21 included patients, three had seizure with migraine as the main phenotype of NIID. However, they did not undergo cerebral perfusion imaging, and the possibility of headache, epilepsy, and NIID cannot be ruled out as independent entities. More cases and further research are required to explore the relationship between these three diseases.

In addition, in comparison with healthy controls, patients with NIID have reduced cortical perfusion but increased deep brain perfusion (9, 40, 41). Characteristic changes in cerebral blood flow in patients with NIID may also cause hypoxia or hypoxic encephalopathy (42–46), which are the most common causes of NCSE (47, 48). In the hyperacute phase of NIID, cerebral hypoperfusion leads to the consumption of oxygen and nutrients, contributing to the accumulation of inclusion bodies in the eosinophilic nucleus and causing cerebral hypoxia, leading to progression followed by hyperperfusion lasting days to weeks (29, 45, 49, 50). After an acute attack, hyperperfusion and vascular hematoma in the cortical area are corrected, and cortical enhancement is alleviated (34). However, because of microvascular injury or intracellular neuronal intranuclear inclusions (NIIs) in subcortical cells after hyperperfusion, which are not conducive to recovery from energy metabolism disorders in the region, sustained chronic ischemia gradually occurs in the cells, leading to cytotoxic edema and angiogenesis, followed by the subcortical ribbon sign (38). DWI exhibits a signal hyperintensity similar to that of ischemic stroke, with significant apoptosis in the region and a pathological presentation of spongiform degeneration (34, 38). Ischemic hypoxic encephalopathy and extensive cytotoxic edema may lead to seizures (47, 48, 51).

We observed that all adult patients with NIID with seizures exhibited diffuse bilateral symmetric white matter lesions on T2WI and FLAIR images. Diffusion tensor imaging (DTI) in Case 9 also revealed sparse white matter fiber bundles (39). Pathological studies have reported extensive white matter fiber disorders throughout the brain of patients with NIID, with decreases in subcortical U-shaped, corpus callosum connective, and pyramidal bundle fibers (52). The causes of white matter lesions are complex. In addition to the influence of changes in cerebral perfusion, the dysfunction of astrocytes with nuclear inclusion bodies may lead to secondary damage to the myelin sheath and axons of the white matter, leading to spongiform degeneration (43), and may also be related to the loss of myelinated nerve fibers and oligodendrocyte degeneration in the white matter (52, 53). In addition, the abnormal amplification of GGC repeat sequences in *NOTCH2NLC* is the main cause of white matter lesions (54). The epileptic focus in patients with NIID is located near the high-signal area at the corticomedullary junction on DWI, suggesting that the nearby cerebral cortex forms the epileptic focus (6). In cases of cerebrovascular disease, concurrent seizures may occur if imaging reveals lesions in the white matter directly below the cortex (6). Therefore, changes in the cortex near the white matter lesions in patients with NIID may be related to seizures.

Among other auxiliary examinations, some patients showed abnormal neuropsychological scores, reflecting abnormalities in frontal lobe function and cognitive impairment. On pathological examination, 20 of the 21 patients showed characteristic eosinophilic inclusion bodies in the nucleus on skin biopsy. Genetic testing revealed abnormal repeated amplification of the GGC sequence in *NOTCH2NLC* in patients with NIID. This amplification is associated with NIID onset (36, 55–58), and *NOTCH2NLC* amplification and mutation are the most common causes of nonvascular white matter lesions. The oligomeric form of the mutated protein (rather than eosinophilic inclusion bodies) is the main pathogenic factor (10, 59–62, 64), and abnormal nuclear aggregation may cause seizures. Further research on NII components could enhance our understanding of the pathophysiology of epilepsy (5, 65–67). Case 13 showed abnormal GGC repeat sequences in *NOTCH2NLC*, without positive pathological and neuroimaging findings, confirming that genetic testing can be a sensitive diagnostic tool (34). The frequency of GGC repeats in the 5′ region of NOTCH2NLC in healthy adults does not exceed 40, and a repeat frequency > 60 is considered pathogenic (14, 68–70). After classification based on the clinical manifestations of NIID, the number of repeats varied among the different subtypes. Patients with myasthenia as the main phenotype had 118–517 repetitions; Parkinson’ s type, 66–102 repetitions; and dementia type, 91–268 repetitions (71, 72). In the present review, six patients with NIID with seizures who underwent genetic testing showed 72–134 GGC sequence repeats. However, the association between these repeats and seizures requires further investigation.

This systematic review had some limitations. First, the included cases were obtained via database search and inclusion and exclusion criteria were applied; therefore, the inclusion of cases may have been biased. Second, due to the rarity of cases of NIID with seizure and incomplete data, only a small portion of NIID cases with seizures were included here (21/39). Therefore, larger multicenter joint studies are needed to confirm the present findings.

In conclusion, NIID is a degenerative disease of the central nervous system that progresses slowly and exhibits heterogeneous clinical manifestations. This systematic review revealed that the most common clinical phenotypes of patients with NIID with seizures were cognitive dysfunction and consciousness disorders, and significant cognitive decline can occur after seizures. This further understanding of the clinical phenotypic characteristics of NIID provides novel insight into early diagnosis in clinical practice. NIID cases exhibit various types of seizures, with the most common being fully originating motor seizures. When compared with infantile and juvenile patients with NIID with seizures, adult patients have a better prognosis and a relatively stable condition. Changes in cortical and medullary perfusion, white matter lesions, abnormal aggregation of gene-mediated proteins, cerebral ischemia, and hypoxia in patients with NIID may be involved in the pathological and physiological mechanisms of seizures. Seizures can lead to secondary brain damage, neurological deficits, disease progression, and poor prognosis. Therefore, further exploration of the interaction mechanism between NIID and seizures is of great significance for making diagnoses, adjusting treatment plans, and improving disease prognosis.

## Data availability statement

The original contributions presented in the study are included in the article/supplementary material, further inquiries can be directed to the corresponding author.

## Author contributions

JZ: Methodology, Software, Supervision, Validation, Writing – original draft, Writing – review & editing. LL: Validation, Writing – review & editing. LX: Supervision, Writing – review & editing. WL: Writing – review & editing. PB: Writing – review & editing. WY: Funding acquisition, Supervision, Validation, Visualization, Writing – review & editing.

## References

[1] Fujita K Osaki Y Miyamoto R Shimatani Y Abe T Sumikura H . Neurologic attack and dynamic perfusion abnormality in neuronal intranuclear inclusion disease. Neurol Clin Pract. (2017) 7:e39–42. doi: 10.1212/cpj.0000000000000389, PMID: 29431160 PMC5800705

[2] Deng J Gu M Miao Y Yao S Zhu M Fang P . Long-read sequencing identified repeat expansions in the 5'UTR of the NOTCH2NLC gene from Chinese patients with neuronal intranuclear inclusion disease. J Med Genet. (2019) 56:758–64. doi: 10.1136/jmedgenet-2019-106268, PMID: 31413119

[3] Yu J Deng J Guo X Shan J Luan X Cao L . The GGC repeat expansion in NOTCH2NLC is associated with oculopharyngodistal myopathy type 3. Brain. (2021) 144:1819–32. doi: 10.1093/brain/awab077, PMID: 33693509 PMC8320266

[4] Fang P Yu Y Yao S Chen S Zhu M Chen Y . Repeat expansion scanning of the NOTCH2NLC gene in patients with multiple system atrophy. Ann Clin Transl Neurol. (2020) 7:517–26. doi: 10.1002/acn3.51021, PMID: 32250060 PMC7187708

[5] Toyota T Huang Z Nohara S Okada K Kakeda S Korogi Y . Neuronal intranuclear inclusion disease manifesting with new-onset epilepsy in the elderly. Neurol Clin Neurosci. (2015) 3:238–40. doi: 10.1111/ncn3.12016

[6] Mao C Zhou L Li J Pang J Chu S Jin W . Clinical-neuroimaging-pathological relationship analysis of adult onset Neuronal Intranuclear Inclusion Disease (NIID). BMC Neurol. (2022) 22:486. doi: 10.1186/s12883-022-03025-1, PMID: 36522621 PMC9753287

[7] Shindo K Tsuchiya M Hata T Ichinose Y Koh K Sone J . Non-convulsive status epilepticus associated with neuronal intranuclear inclusion disease: A case report and literature review. Epilepsy Behav Case Rep. (2019) 11:103–6. doi: 10.1016/j.ebcr.2019.01.007, PMID: 30891404 PMC6403408

[8] Espay AJ Paviour DC O'Sullivan JD Schmidt RE Revilla FJ Metman LV. Juvenile levodopa-responsive Parkinsonism with early orobuccolingual dyskinesias and cognitive impairment. Mov Disord. (2010) 25:1860–7. doi: 10.1002/mds.23194, PMID: 20669183

[9] Sone J Mori K Inagaki T Katsumata R Takagi S Yokoi S . Clinicopathological features of adult-onset neuronal intranuclear inclusion disease. Brain. (2016) 139:3170–86. doi: 10.1093/brain/aww249, PMID: 27797808 PMC5382941

[10] Li M Li K Li X Tian Y Shen L Wu G . Multiple reversible encephalitic attacks: a rare manifestation of neuronal intranuclear inclusion disease. BMC Neurol. (2020) 20:125. doi: 10.1186/s12883-020-01712-5, PMID: 32268889 PMC7140360

[11] Ishiura H Shibata S Yoshimura J Suzuki Y Qu W Doi K . Noncoding CGG repeat expansions in neuronal intranuclear inclusion disease, oculopharyngodistal myopathy and an overlapping disease. Nat Genet. (2019) 51:1222–32. doi: 10.1038/s41588-019-0458-z, PMID: 31332380

[12] Takahashi-Fujigasaki J . Neuronal intranuclear hyaline inclusion disease. Neuropathology. (2003) 23:351–9. doi: 10.1046/j.1440-1789.2003.00524.x 14719553

[13] Lu X Hong D. Neuronal intranuclear inclusion disease: recognition and update. J Neural Transm (Vienna). (2021) 128:295–303. doi: 10.1007/s00702-021-02313-3, PMID: 33599827

[14] Liu Y Li H Liu X Wang B Yang H Wan B . Clinical and mechanism advances of neuronal intranuclear inclusion disease. Front Aging Neurosci. (2022) 14:934725. doi: 10.3389/fnagi.2022.934725, PMID: 36177481 PMC9513122

[15] Mori F Tanji K Kon T Odagiri S Hattori M Hoshikawa Y . FUS immunoreactivity of neuronal and glial intranuclear inclusions in intranuclear inclusion body disease. Neuropathol Appl Neurobiol. (2012) 38:322–8. doi: 10.1111/j.1365-2990.2011.01217.x, PMID: 21883376

[16] Funata N Maeda Y Koike M Yano Y Kaseda M Muro T . Neuronal intranuclear hyaline inclusion disease: report of a case and review of the literature. Clin Neuropathol. (1990) 9:89–96. PMID: 1692776

[17] Zannolli R Gilman S Rossi S Volpi N Bernini A Galluzzi P . Hereditary neuronal intranuclear inclusion disease with autonomic failure and cerebellar degeneration. Arch Neurol. (2002) 59:1319–26. doi: 10.1001/archneur.59.8.1319, PMID: 12164731

[18] Vermilion J Johnson M Srinivasan J Mink JW. Neuronal Intranuclear Inclusion Disease: Longitudinal Case Report of Motor and Nonmotor Symptoms. J Child Neurol. (2019) 34:801–5. doi: 10.1177/0883073819860566, PMID: 31304825 PMC6801045

[19] Sloane AE Becker LE Ang LC Wark J Haslam RH. Neuronal intranuclear hyaline inclusion disease with progressive cerebellar ataxia. Pediatr Neurol. (1994) 10:61–6. doi: 10.1016/0887-8994(94)90070-1, PMID: 7515242

[20] Yamanaka H Hashimoto S Suenaga T. Neuronal intranuclear inclusion disease with prolonged impaired consciousness and status epilepticus: a case report. Rinsho Shinkeigaku. (2019) 59:425–30. doi: 10.5692/clinicalneurol.cn-001264, PMID: 31243248

[21] Liang Y Xu B Xiang G Qi L. Clinical analysis of 6 cases of adult-onset neuronal intranuclear inclusion disease. Chin J Integr Med Cardio-/Cerebrovasc Dis. (2022) 20:1145–8. doi: 10.12102/j.issn.1672-1349.2022.06.048

[22] Fisher RS Cross JH French JA Higurashi N Hirsch E Jansen FE . Operational classification of seizure types by the International League Against Epilepsy: Position Paper of the ILAE Commission for Classification and Terminology. Epilepsia. (2017) 58:522–30. doi: 10.1111/epi.13670, PMID: 28276060

[23] Jia X Li Q Peng Y Zhang S Huang Y Du A. Neuronal intranuclear inclusion disease with mental disorder as the first symptom: a case report and literaure review. J Neurosci Ment Health. (2023) 23:149–52. doi: 10.3969/j.issn.1009-6574.2023.02.013

[24] Lu M Mei S Wu X Xu E. Clinical analysis of sporadic neuronal inclusion body disease in adults. Chin J Practical Nervous Dis. (2022) 25:415–20. doi: 10.12083/SYSJ.220117

[25] Yue B Li L Wang Y Jiang Z. Neuronal intranuclear inclusion disease with symptoms of encephalitis: a case report. J Chin Med Univ. (2021) 50:84–8. doi: 10.12007/j.issn.0258-4646.2021.01.017

[26] Haltia M Somer H Palo J Johnson WG. Neuronal intranuclear inclusion disease in identical twins. Ann Neurol. (1984) 15:316–21. doi: 10.1002/ana.410150403, PMID: 6331275

[27] Guo JJ Wang ZY Wang M Jiang ZZ Yu XF. Neuronal intranuclear inclusion disease mimicking acute cerebellitis: A case report. World J Clin Cases. (2020) 8:6122–9. doi: 10.12998/wjcc.v8.i23.6122, PMID: 33344613 PMC7723690

[28] Ueda R Koizumi T Mizuno T Nakagawa M. Neuronal intranuclear inclusion disease in a patient who exhibited abnormal behavior. Rinsho Shinkeigaku. (2022) 62:369–74. doi: 10.5692/clinicalneurol.cn-001689, PMID: 35474285

[29] Ataka T Kimura N Matsubara E. Temporal Changes in Brain Perfusion in Neuronal Intranuclear Inclusion Disease. Intern Med. (2021) 60:941–4. doi: 10.2169/internalmedicine.5743-20, PMID: 33087670 PMC8024962

[30] Biton V Gates JR dePadua SL. Prolonged postictal encephalopathy. Neurology. (1990) 40:963–6. doi: 10.1212/wnl.40.6.963 2345618

[31] Donaire A Carreno M Gómez B Fossas P Bargalló N Agudo R . Cortical laminar necrosis related to prolonged focal status epilepticus. J Neurol Neurosurg Psychiatry. (2006) 77:104–6. doi: 10.1136/jnnp.2004.058701, PMID: 16361606 PMC2117425

[32] Giovannini G Kuchukhidze G McCoy MR Meletti S Trinka E. Neuroimaging alterations related to status epilepticus in an adult population: Definition of MRI findings and clinical-EEG correlation. Epilepsia. (2018) 59:120–7. doi: 10.1111/epi.14493 30129213

[33] Men S Lee DH Barron JR Muñoz DG. Selective neuronal necrosis associated with status epilepticus: MR findings. AJNR Am J Neuroradiol. (2000) 21:1837–40. PMID: 11110535 PMC7974287

[34] Cao Y Wu J Yue Y Zhang C Liu S Zhong P . Expanding the clinical spectrum of adult-onset neuronal intranuclear inclusion disease. Acta Neurol Belg. (2022) 122:647–58. doi: 10.1007/s13760-021-01622-4, PMID: 33625684

[35] Yadav N Raja P Shetty SS Jitender S Prasad C Kamble NL . Neuronal Intranuclear Inclusion Disease: A Rare Etiology for Rapidly Progressive Dementia. Alzheimer Dis Assoc Disord. (2019) 33:359–61. doi: 10.1097/wad.0000000000000312 31094708

[36] Abe K Fujita M. Over 10 years MRI observation of a patient with neuronal intranuclear inclusion disease. BMJ Case Rep. (2017) 2017:bcr2016218790. doi: 10.1136/bcr-2016-218790, PMID: 28237949 PMC5337643

[37] Wang Y Wang B Wang L Yao S Zhao J Zhong S . Diagnostic indicators for adult-onset neuronal intranuclear inclusion disease. Clin Neuropathol. (2020) 39:7–18. doi: 10.5414/np301203, PMID: 31661069

[38] Araki K Sone J Fujioka Y Masuda M Ohdake R Tanaka Y . Memory Loss and Frontal Cognitive Dysfunction in a Patient with Adult-onset Neuronal Intranuclear Inclusion Disease. Intern Med. (2016) 55:2281–4. doi: 10.2169/internalmedicine.55.5544, PMID: 27523009

[39] Huang W Li X Wang J Fan F Zhang P Zhang J. MRI manifestations of adult-onset neuronal intranuclear inclusion disease. Chin J Med Imaging Technol. (2021) 37:1281–5. doi: 10.13929/j.issn.1003-3289.2021.09.001

[40] Tai HF Hua TT Zhang ZQ Duan YY Zhuo ZZ Wang A . Characteristic cerebral perfusion pattern in neuronal intranuclear inclusion disease. Front Neurosci. (2022) 16:1081383. doi: 10.3389/fnins.2022.1081383, PMID: 36570826 PMC9768440

[41] Blanco PJ Müller LO Spence JD. Blood pressure gradients in cerebral arteries: a clue to pathogenesis of cerebral small vessel disease. Stroke Vasc Neurol. (2017) 2:108–17. doi: 10.1136/svn-2017-000087, PMID: 28989801 PMC5628379

[42] Yoshii D Ayaki T Wada T Ozaki A Yamamoto T Miyagi Y . An autopsy case of adult-onset neuronal intranuclear inclusion disease with perivascular preservation in cerebral white matter. Neuropathology. (2022) 42:66–73. doi: 10.1111/neup.12778, PMID: 34954850

[43] Orihara A Miyakoshi N Sunami Y Kimura H Nakata Y Komori T . Acute Reversible Encephalopathy with Neuronal Intranuclear Inclusion Disease Diagnosed by a Brain Biopsy: Inferring the Mechanism of Encephalopathy from Radiological and Histological Findings. Intern Med. (2023) 62:1821–5. doi: 10.2169/internalmedicine.0156-22, PMID: 36288982 PMC10332957

[44] Oláhová M Hardy SA Hall J Yarham JW Haack TB Wilson WC . LRPPRC mutations cause early-onset multisystem mitochondrial disease outside of the French-Canadian population. Brain. (2015) 138:3503–19. doi: 10.1093/brain/awv291, PMID: 26510951 PMC4655343

[45] Siira SJ Spåhr H Shearwood AJ Ruzzenente B Larsson NG Rackham O . LRPPRC-mediated folding of the mitochondrial transcriptome. Nat Commun. (2017) 8:1532. doi: 10.1038/s41467-017-01221-z, PMID: 29146908 PMC5691074

[46] Gueven N Woolley K Smith J. Border between natural product and drug: comparison of the related benzoquinones idebenone and coenzyme Q10. Redox Biol. (2015) 4:289–95. doi: 10.1016/j.redox.2015.01.009, PMID: 25625583 PMC4803797

[47] Meierkord H Holtkamp M. Non-convulsive status epilepticus in adults: clinical forms and treatment. Lancet Neurol. (2007) 6:329–39. doi: 10.1016/s1474-4422(07)70074-1 17362837

[48] Maganti R Gerber P Drees C Chung S. Nonconvulsive status epilepticus. Epilepsy Behav. (2008) 12:572–86. doi: 10.1016/j.yebeh.2007.12.002 18248774

[49] Wang R Nie X Xu S Zhang M Dong Z Yu S. Interrelated Pathogenesis? Neuronal Intranuclear Inclusion Disease Combining With Hemiplegic Migraine. Headache. (2020) 60:382–95. doi: 10.1111/head.13687, PMID: 31701545

[50] Yu J Liufu T Zheng Y Xu J Meng L Zhang W . CGG repeat expansion in NOTCH2NLC causes mitochondrial dysfunction and progressive neurodegeneration in Drosophila model. Proc Natl Acad Sci USA. (2022) 119:e2208649119. doi: 10.1073/pnas.2208649119, PMID: 36191230 PMC9565157

[51] Sui Y Shi Y Yang Y Lv Z. Neuronal intranuclear inclusion disease: a case report and literature review. Acta Neurol Belg. (2023) 123:1651–3. doi: 10.1007/s13760-023-02210-4, PMID: 36897506

[52] Yokoi S Yasui K Hasegawa Y Niwa K Noguchi Y Tsuzuki T . Pathological background of subcortical hyperintensities on diffusion-weighted images in a case of neuronal intranuclear inclusion disease. Clin Neuropathol. (2016) 35:375–80. doi: 10.5414/np300961 27719745

[53] Zhou Y Huang P Huang Z Peng Y Zheng Y Yu Y . Urine cytological study in patients with clinicopathologically confirmed neuronal intranuclear inclusion disease. Front Aging Neurosci. (2022) 14:977604. doi: 10.3389/fnagi.2022.977604, PMID: 36172483 PMC9510843

[54] Liu YH Chou YT Chang FP Lee WJ Guo YC Chou CT . Neuronal intranuclear inclusion disease in patients with adult-onset non-vascular leukoencephalopathy. Brain. (2022) 145:3010–21. doi: 10.1093/brain/awac135, PMID: 35411397

[55] Tian Y Wang JL Huang W Zeng S Jiao B Liu Z . Expansion of Human-Specific GGC Repeat in Neuronal Intranuclear Inclusion Disease-Related Disorders. Am J Hum Genet. (2019) 105:166–76. doi: 10.1016/j.ajhg.2019.05.013, PMID: 31178126 PMC6612530

[56] Suzuki IK Gacquer D Van Heurck R Kumar D Wojno M Bilheu A . Human-Specific NOTCH2NL Genes Expand Cortical Neurogenesis through Delta/Notch Regulation. Cell. (2018) 173:1370–84.e16. doi: 10.1016/j.cell.2018.03.067, PMID: 29856955 PMC6092419

[57] Fiddes IT Lodewijk GA Mooring M Bosworth CM Ewing AD Mantalas GL . Human-Specific NOTCH2NL Genes Affect Notch Signaling and Cortical Neurogenesis. Cell. (2018) 173:1356–69.e22. doi: 10.1016/j.cell.2018.03.051, PMID: 29856954 PMC5986104

[58] Tian Y Zhou L Gao J Jiao B Zhang S Xiao Q . Clinical features of NOTCH2NLC-related neuronal intranuclear inclusion disease. J Neurol Neurosurg Psychiatry. (2022) 93:1289–98. doi: 10.1136/jnnp-2022-329772, PMID: 36150844 PMC9685690

[59] Zhao B Yang M Wang Z Yang Q Zhang Y Qi X . Clinical characteristics of two patients with neuronal intranuclear inclusion disease and literature review. Front Neurosci. (2022) 16:1056261. doi: 10.3389/fnins.2022.1056261, PMID: 36545534 PMC9762495

[60] Liu Q Zhang K Kang Y Li Y Deng P Li Y . Expression of expanded GGC repeats within NOTCH2NLC causes behavioral deficits and neurodegeneration in a mouse model of neuronal intranuclear inclusion disease. Sci Adv. (2022) 8:eadd6391. doi: 10.1126/sciadv.add6391, PMID: 36417528 PMC9683706

[61] Huang XR Tang BS Jin P Guo JF. The Phenotypes and Mechanisms of NOTCH2NLC-Related GGC Repeat Expansion Disorders: a Comprehensive Review. Mol Neurobiol. (2022) 59:523–34. doi: 10.1007/s12035-021-02616-2, PMID: 34718964

[62] Glineburg MR Todd PK Charlet-Berguerand N Sellier C. Repeat-associated non-AUG (RAN) translation and other molecular mechanisms in Fragile X Tremor Ataxia Syndrome. Brain Res. (2018) 1693:43–54. doi: 10.1016/j.brainres.2018.02.006, PMID: 29453961 PMC6010627

[63] Patel H Norman MG Perry TL Berry KE. Multiple system atrophy with neuronal intranuclear hyaline inclusions. Report of a case and review of the literature. J Neurol Sci. (1985) 67:57–65. doi: 10.1016/0022-510x(85)90022-x, PMID: 2580060

[64] Ehrlich ME Ellerby LM. Neuronal intranuclear inclusion disease: Polyglycine protein is the culprit. Neuron. (2021) 109:1757–60. doi: 10.1016/j.neuron.2021.05.018, PMID: 34081916 PMC10773977

[65] Doi H Adachi H Katsuno M Minamiyama M Matsumoto S Kondo N . p62/SQSTM1 differentially removes the toxic mutant androgen receptor via autophagy and inclusion formation in a spinal and bulbar muscular atrophy mouse model. J Neurosci. (2013) 33:7710–27. doi: 10.1523/jneurosci.3021-12.2013 23637164 PMC6618982

[66] Park H Yamanaka T Toyama Y Fujita A Doi H Nirasawa T . Hornerin deposits in neuronal intranuclear inclusion disease: direct identification of proteins with compositionally biased regions in inclusions. Acta Neuropathol Commun. (2022) 10:28. doi: 10.1186/s40478-022-01333-8, PMID: 35246273 PMC8895595

[67] Nakano Y Takahashi-Fujigasaki J Sengoku R Kanemaru K Arai T Kanda T . PML Nuclear Bodies Are Altered in Adult-Onset Neuronal Intranuclear Hyaline Inclusion Disease. J Neuropathol Exp Neurol. (2017) 76:585–94. doi: 10.1093/jnen/nlx039, PMID: 28863453

[68] Sun QY Xu Q Tian Y Hu ZM Qin LX Yang JX . Expansion of GGC repeat in the human-specific NOTCH2NLC gene is associated with essential tremor. Brain. (2020) 143:222–33. doi: 10.1093/brain/awz372, PMID: 31819945

[69] Deng J Zhou B Yu J Han X Fu J Li X . Genetic origin of sporadic cases and RNA toxicity in neuronal intranuclear inclusion disease. J Med Genet. (2022) 59:462–9. doi: 10.1136/jmedgenet-2020-107649, PMID: 33766934

[70] Barbé L Lanni S López-Castel A Franck S Spits C Keymolen K . CpG Methylation, a Parent-of-Origin Effect for Maternal-Biased Transmission of Congenital Myotonic Dystrophy. Am J Hum Genet. (2017) 100:488–505. doi: 10.1016/j.ajhg.2017.01.033, PMID: 28257691 PMC5339342

[71] Sone J Mitsuhashi S Fujita A Mizuguchi T Hamanaka K Mori K . Long-read sequencing identifies GGC repeat expansions in NOTCH2NLC associated with neuronal intranuclear inclusion disease. Nat Genet. (2019) 51:1215–21. doi: 10.1038/s41588-019-0459-y, PMID: 31332381

[72] Ma D Tan YJ Ng ASL Ong HL Sim W Lim WK . Association of NOTCH2NLC Repeat Expansions With Parkinson Disease. JAMA Neurol. (2020) 77:1559–63. doi: 10.1001/jamaneurol.2020.3023, PMID: 32852534 PMC7445625

